# Supplementary material for: Immediate recruitment of dormant coronary collaterals can provide more than half of normal resting perfusion during coronary occlusion in patients with coronary artery disease
Source: J Nucl Cardiol. 2023 Jun 6;30(6):2338–45. doi: 10.1007/s12350-023-03271-x (PMC10682227; doi:10.1007/s12350-023-03271-x)
Supplement: Supplementary file 1 — Supplementary file1 (PPTX 979 kb) [file 12350_2023_3271_MOESM1_ESM.pptx]

## Slide 1
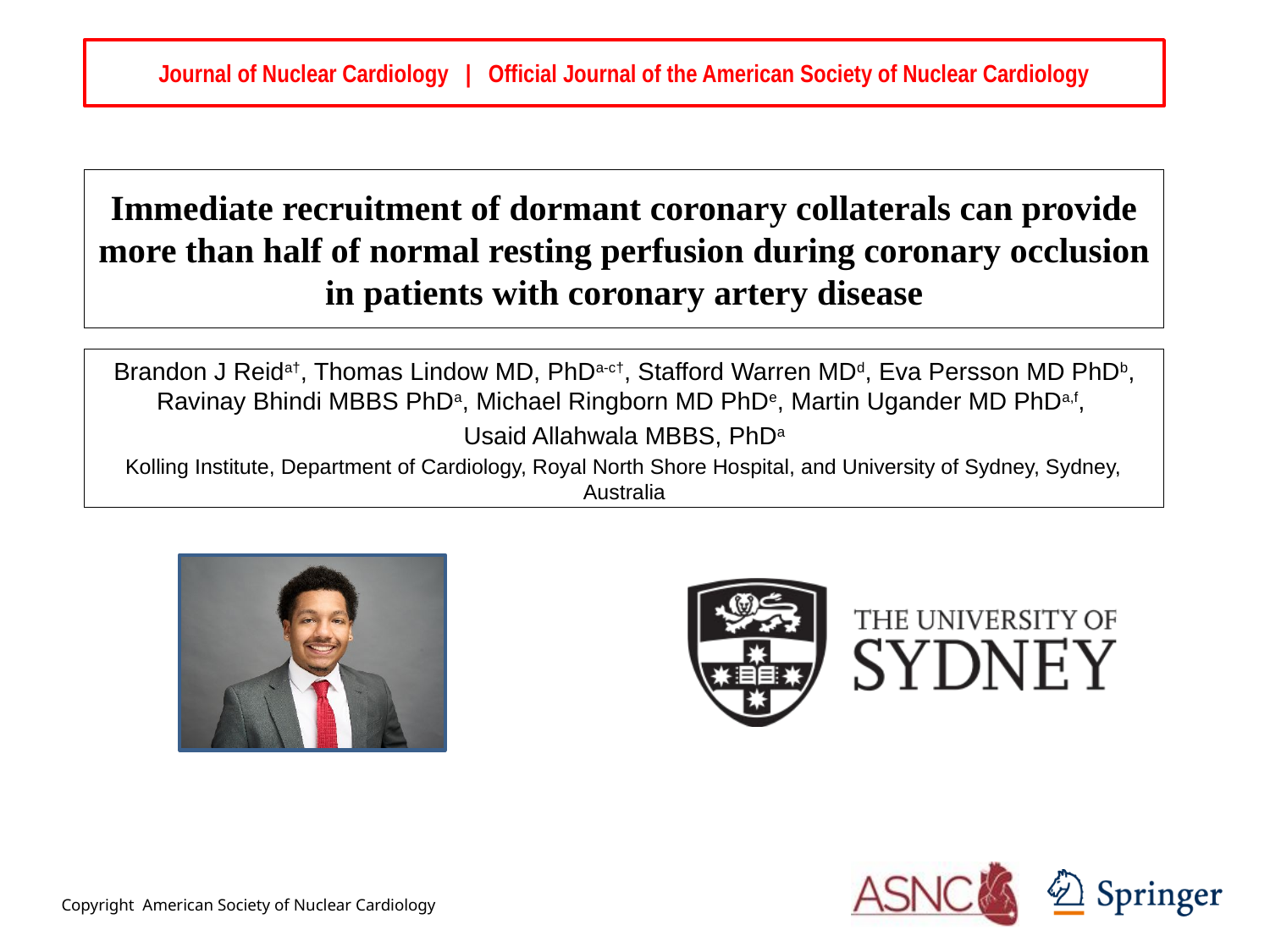

Journal of Nuclear Cardiology | Official Journal of the American Society of Nuclear Cardiology
# Immediate recruitment of dormant coronary collaterals can provide more than half of normal resting perfusion during coronary occlusion in patients with coronary artery disease
Brandon J Reida†, Thomas Lindow MD, PhDa-c†, Stafford Warren MDd, Eva Persson MD PhDb, Ravinay Bhindi MBBS PhDa, Michael Ringborn MD PhDe, Martin Ugander MD PhDa,f,
Usaid Allahwala MBBS, PhDa
Kolling Institute, Department of Cardiology, Royal North Shore Hospital, and University of Sydney, Sydney, Australia
Copyright American Society of Nuclear Cardiology

## Slide 2
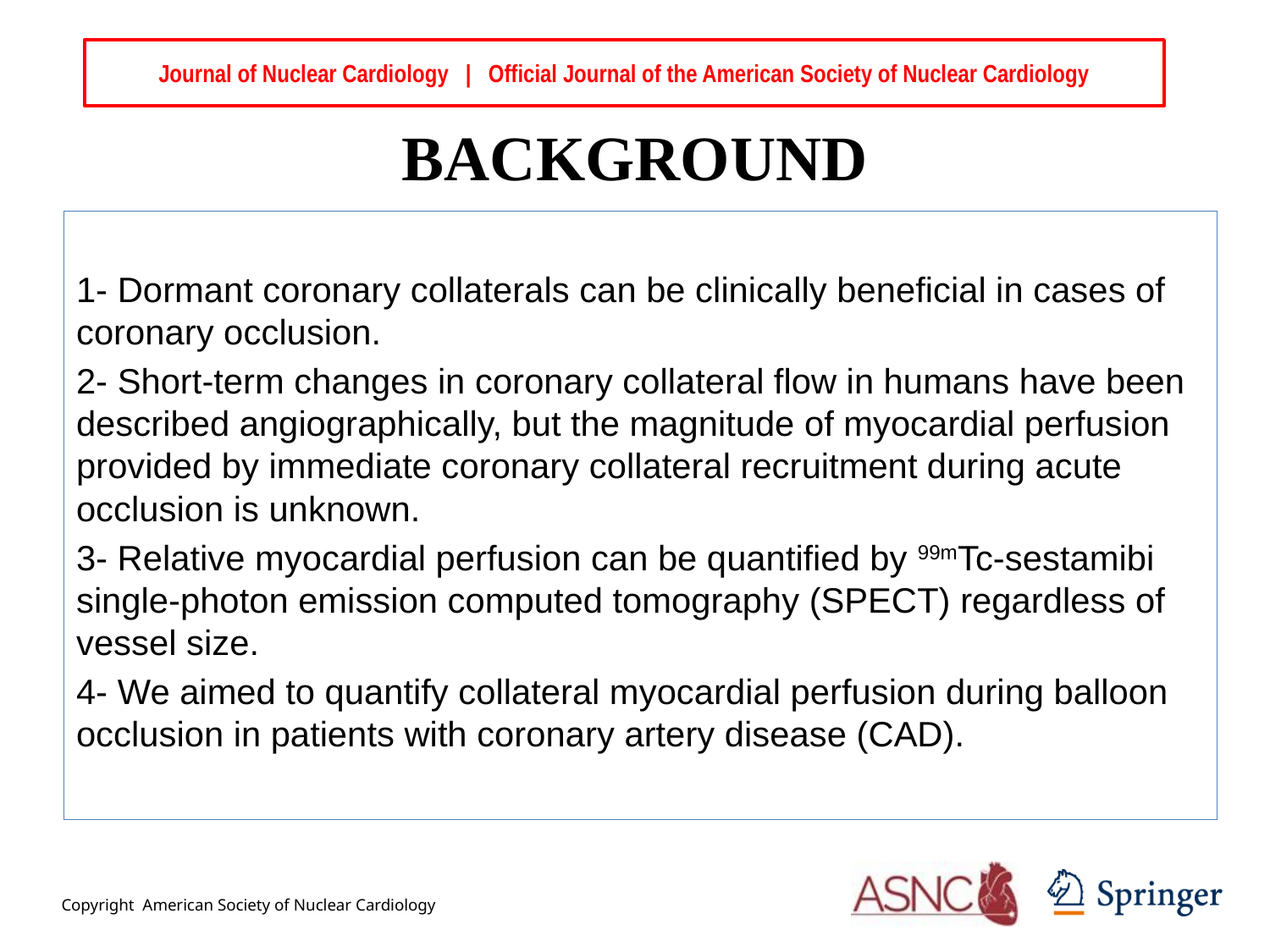

Journal of Nuclear Cardiology | Official Journal of the American Society of Nuclear Cardiology
# BACKGROUND
1- Dormant coronary collaterals can be clinically beneficial in cases of coronary occlusion.
2- Short-term changes in coronary collateral flow in humans have been described angiographically, but the magnitude of myocardial perfusion provided by immediate coronary collateral recruitment during acute occlusion is unknown.
3- Relative myocardial perfusion can be quantified by 99mTc-sestamibi single-photon emission computed tomography (SPECT) regardless of vessel size.
4- We aimed to quantify collateral myocardial perfusion during balloon occlusion in patients with coronary artery disease (CAD).
Copyright American Society of Nuclear Cardiology

## Slide 3
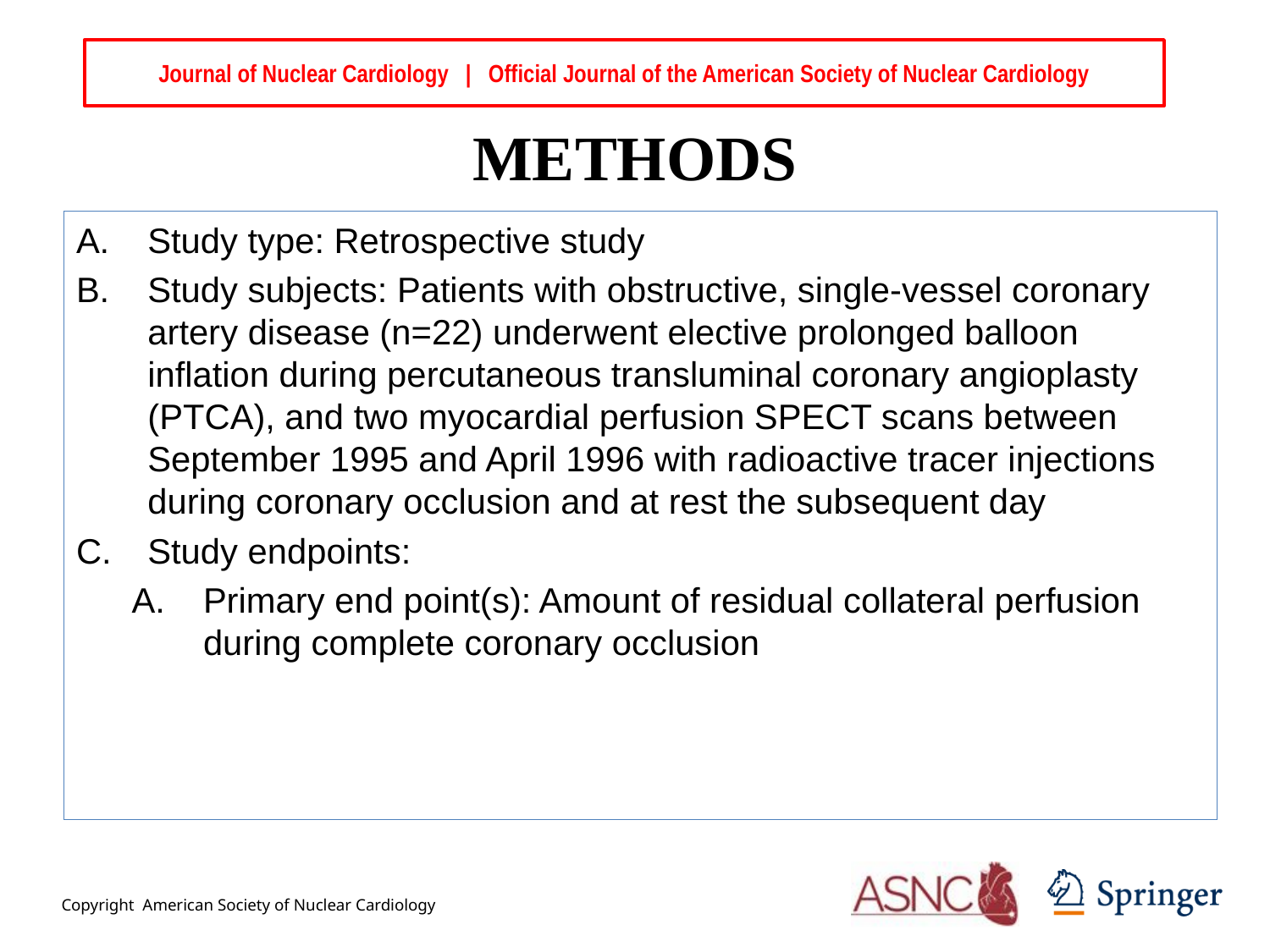

Journal of Nuclear Cardiology | Official Journal of the American Society of Nuclear Cardiology
# METHODS
Study type: Retrospective study
Study subjects: Patients with obstructive, single-vessel coronary artery disease (n=22) underwent elective prolonged balloon inflation during percutaneous transluminal coronary angioplasty (PTCA), and two myocardial perfusion SPECT scans between September 1995 and April 1996 with radioactive tracer injections during coronary occlusion and at rest the subsequent day
Study endpoints:
Primary end point(s): Amount of residual collateral perfusion during complete coronary occlusion
Copyright American Society of Nuclear Cardiology

## Slide 4
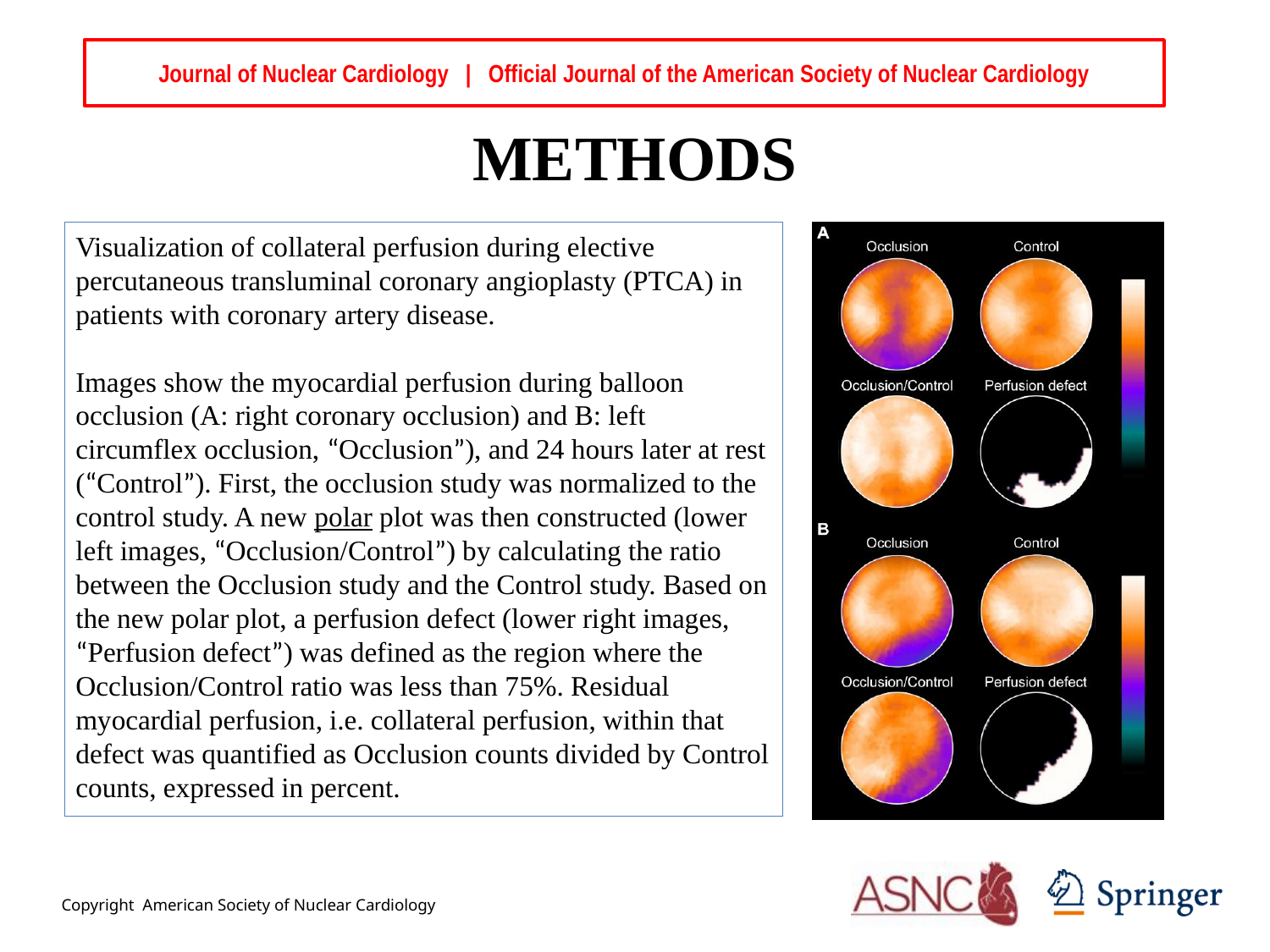

Journal of Nuclear Cardiology | Official Journal of the American Society of Nuclear Cardiology
# METHODS
Visualization of collateral perfusion during elective percutaneous transluminal coronary angioplasty (PTCA) in patients with coronary artery disease.
Images show the myocardial perfusion during balloon occlusion (A: right coronary occlusion) and B: left circumflex occlusion, “Occlusion”), and 24 hours later at rest (“Control”). First, the occlusion study was normalized to the control study. A new polar plot was then constructed (lower left images, “Occlusion/Control”) by calculating the ratio between the Occlusion study and the Control study. Based on the new polar plot, a perfusion defect (lower right images, “Perfusion defect”) was defined as the region where the Occlusion/Control ratio was less than 75%. Residual myocardial perfusion, i.e. collateral perfusion, within that defect was quantified as Occlusion counts divided by Control counts, expressed in percent.
Copyright American Society of Nuclear Cardiology

## Slide 5
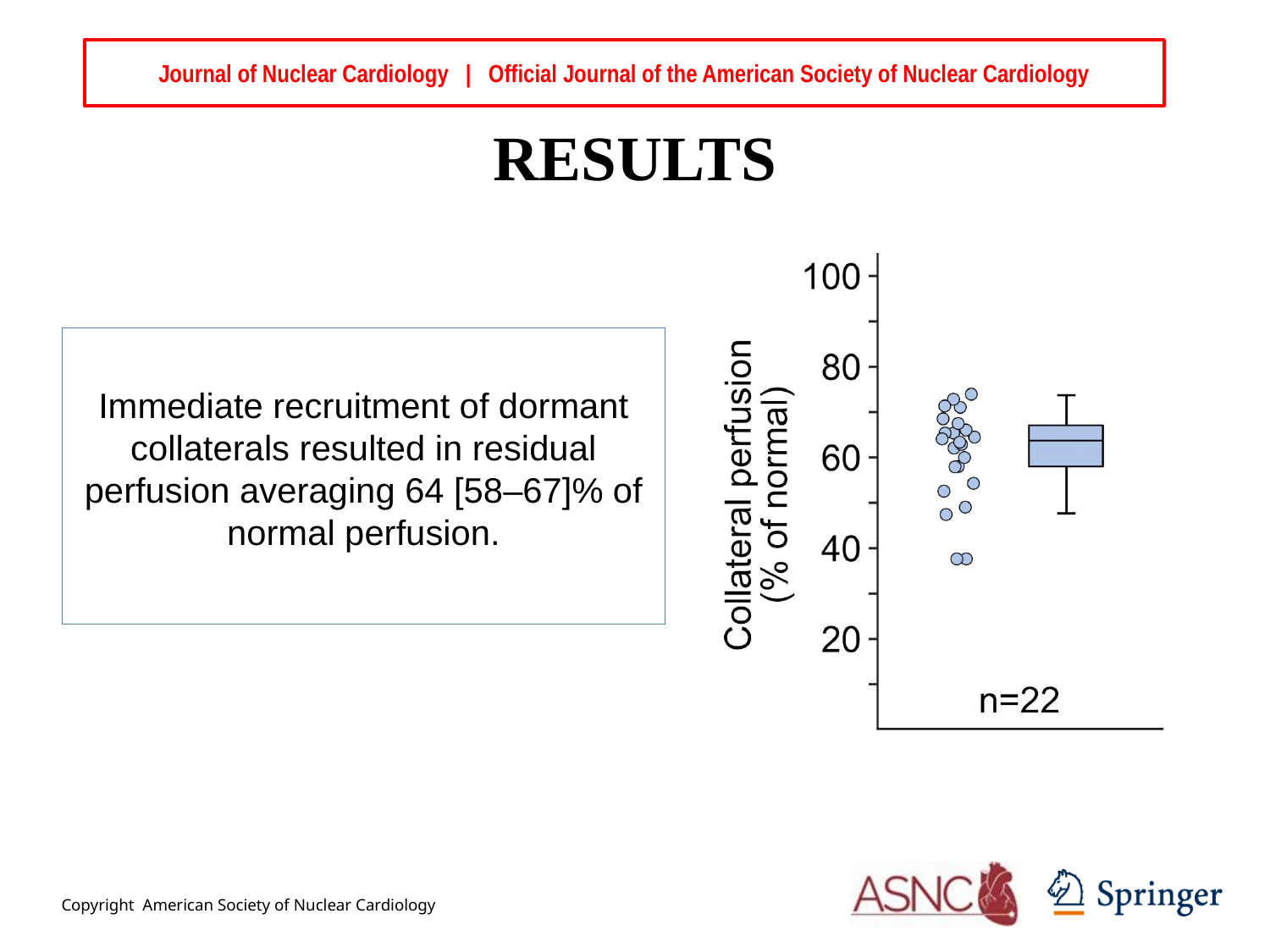

Journal of Nuclear Cardiology | Official Journal of the American Society of Nuclear Cardiology
# RESULTS
Immediate recruitment of dormant collaterals resulted in residual perfusion averaging 64 [58–67]% of normal perfusion.
Copyright American Society of Nuclear Cardiology

## Slide 6
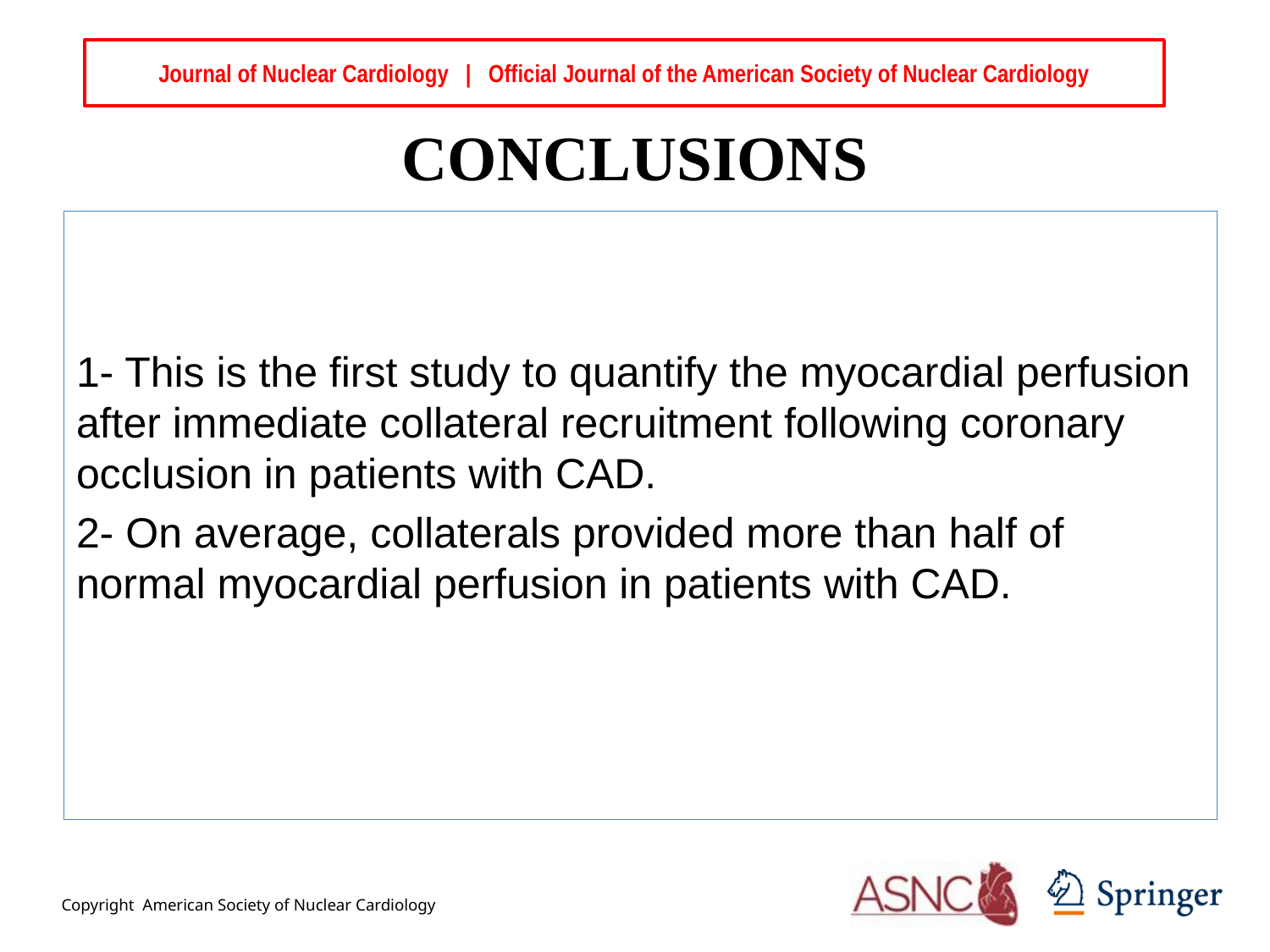

Journal of Nuclear Cardiology | Official Journal of the American Society of Nuclear Cardiology
# CONCLUSIONS
1- This is the first study to quantify the myocardial perfusion after immediate collateral recruitment following coronary occlusion in patients with CAD.
2- On average, collaterals provided more than half of normal myocardial perfusion in patients with CAD.
Copyright American Society of Nuclear Cardiology
