# Supplementary material for: Immediate recruitment of dormant coronary collaterals can provide more than half of normal resting perfusion during coronary occlusion in patients with coronary artery disease
Source: J Nucl Cardiol. 2023 Jun 6;30(6):2338–45. doi: 10.1007/s12350-023-03271-x (PMC10682227; doi:10.1007/s12350-023-03271-x)
Supplement: Supplementary file 2 — Supplementary file2 (PPTX 34 kb) [file 12350_2023_3271_MOESM2_ESM.pptx]

## Slide 1
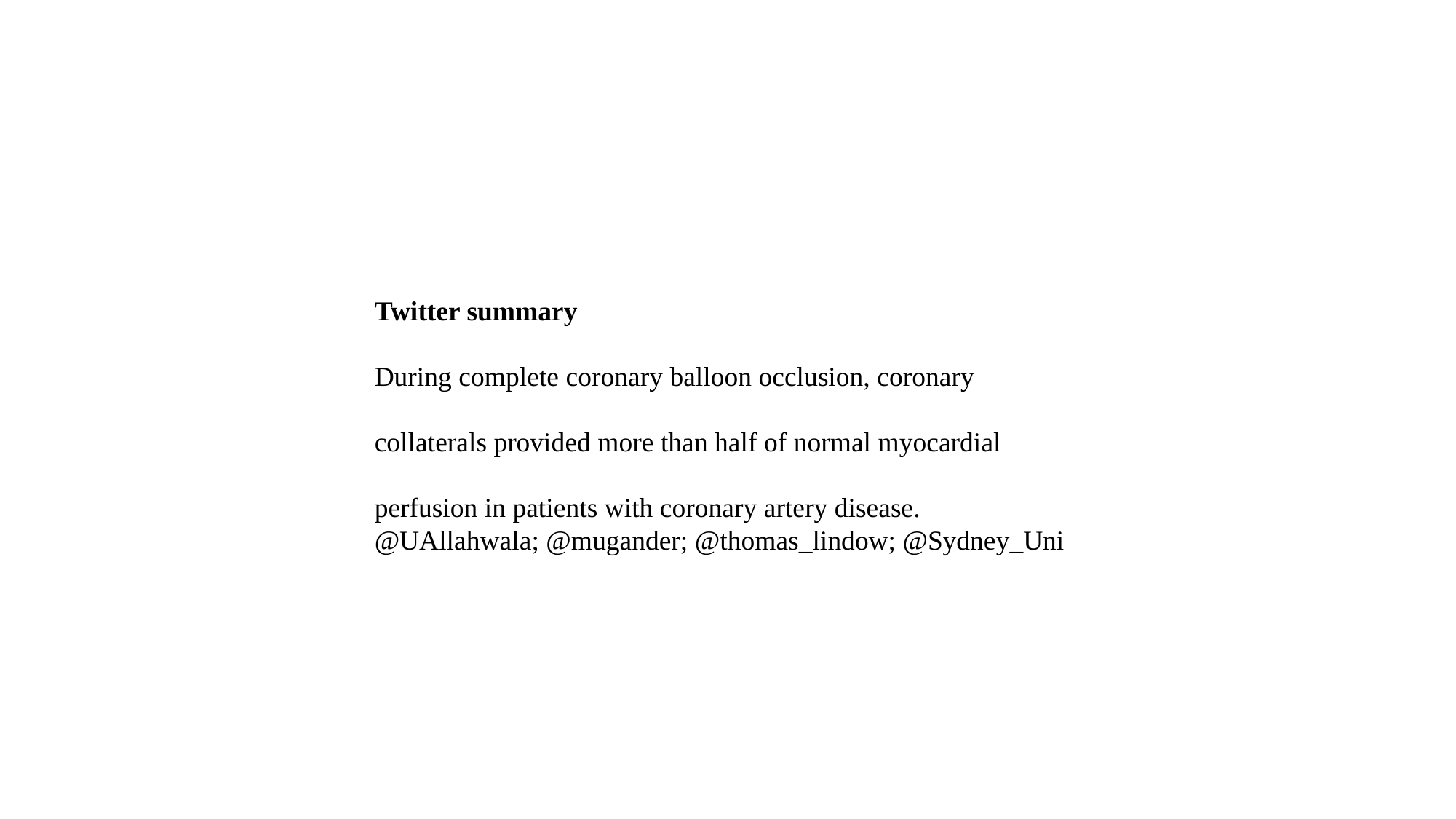

Twitter summary
During complete coronary balloon occlusion, coronary collaterals provided more than half of normal myocardial perfusion in patients with coronary artery disease.
@UAllahwala; @mugander; @thomas_lindow; @Sydney_Uni
